# Co-Inference of Data Mislabelings Reveals Improved Models in Genomics and Breast Cancer Diagnostics

**DOI:** 10.3389/frai.2021.739432

**Published:** 2022-01-05

**Authors:** Susanne Gerber, Lukas Pospisil, Stanislav Sys, Charlotte Hewel, Ali Torkamani, Illia Horenko

**Affiliations:** ^1^ Institute of Human Genetics, University Medical Center of the Johannes Gutenberg University Mainz, Mainz, Germany; ^2^ Faculty of Informatics, Institute of Computational Science, Università Della Svizzera Italiana, Lugano, Switzerland; ^3^ Department of Integrative Structural and Computational Biology, The Scripps Research Institute, La Jolla, CA, United States

**Keywords:** mislabeling, label noise, latent variable estimation, bioinformatics, bias, regression, machine learning

## Abstract

Mislabeling of cases as well as controls in case–control studies is a frequent source of strong bias in prognostic and diagnostic tests and algorithms. Common data processing methods available to the researchers in the biomedical community do not allow for consistent and robust treatment of labeled data in the situations where both, the case and the control groups, contain a non-negligible proportion of mislabeled data instances. This is an especially prominent issue in studies regarding late-onset conditions, where individuals who may convert to cases may populate the control group, and for screening studies that often have high false-positive/-negative rates. To address this problem, we propose a method for a simultaneous robust inference of Lasso reduced discriminative models and of latent group-specific mislabeling risks, not requiring any exactly labeled data. We apply it to a standard breast cancer imaging dataset and infer the mislabeling probabilities (being rates of false-negative and false-positive core-needle biopsies) together with a small set of simple diagnostic rules, outperforming the state-of-the-art BI-RADS diagnostics on these data. The inferred mislabeling rates for breast cancer biopsies agree with the published purely empirical studies. Applying the method to human genomic data from a healthy-ageing cohort reveals a previously unreported compact combination of single-nucleotide polymorphisms that are strongly associated with a healthy-ageing phenotype for Caucasians. It determines that 7.5*%* of Caucasians in the 1000 Genomes dataset (selected as a control group) carry a pattern characteristic of healthy ageing.

## Introduction

The analysis of biomedical data often aims to identify a specific (small) set of characteristics or biomarkers that will allow for the most accurate and efficient discrimination between groups. For example, it is assumed that an unknown combination of characteristics offers the best possibility to distinguish a group of patients with a certain symptom or disease from all other groups. A wide variety of statistical and machine learning tools have been developed to select the optimal feature set through an analysis of labeled data (Bair and Tibshirani, 2004; Hastie et al., 2009; Luo and Ren, 2014; Taylor and Tibshirani, 2015). In practice, however, mistakes in the assignment of labels and features during the data acquisition procedure may introduce serious bias when common methods are applied or even prohibit their use. Erroneous assignments occur in many studies for a variety of reasons: experimental errors, differences in platforms used to acquire data from different groups, differences in protocols used to post-process data, or intrinsic difficulties in distinguishing the case and control groups (Lam et al., 2011; ORawe et al., 2013; Ross et al., 2013; Weißbach et al., 2021). This is particularly evident in the case of coronavirus data emerging from different sources where it has been shown that data variability is an important factor concerning the usability of such data for machine learning (Sáez et al., 2020). Furthermore, diagnoses for many diseases only reach a certain level of confidence; in some cases (e.g., Alzheimer’s disease), a 100% diagnosis is only possible during a postmortem autopsy (Gomez-Nicola and Boche, 2015). This means that subjects with a negative diagnosis might nevertheless carry a latent form of a disease without showing symptoms yet. Assigning those individuals to the control group in a (bio)medical study can introduce a strong source of errors and can certainly have severe consequences for the patients, if this mislabeling cannot be identified, and correct medical treatment will be withheld.

Most methods that are commonly applied to deal with the problem of mislabeling are based on detection of anomalies and outliers and aim to avoid the problem by thoroughly cleaning the data during preprocessing, removing those points that appear to be mislabeled (Brodley and Friedl, 1999; Barandela and Gasca, 2000; Teng, 2001; Jiang and Zhou, 2004; Frénay and Kaban, 2014; Frenay and Verleysen, 2014). They detect outliers because they deviate significantly from a model that has already been imposed on a data subset—which is again—presumed to be correctly labeled (Chandola et al., 2009). This cleaning however can be problematic because it first may not be possible if too little is known to determine what might be mislabeled, and second because it can severely reduce the size of the available data, making statistical results less reliable (Teng, 2001; Bootkrajang and Kabán, 2012). Popular methods to analyze data based on supervised (Tibshirani, 1996; Hastie et al., 2009; Luo and Ren, 2014) and semi-supervised machine learning methods (Moya and Hush, 1996; Bair and Tibshirani, 2004; Rodionova et al., 2016) for labeled data analysis (e.g., generalized linear models and neuronal networks) are also implicitly based on an assumption that at least one of the groups to be discriminated has been labeled perfectly or at least assume a subset of perfectly labeled data (Hendrycks et al., 2019). On the other side, common *unsupervised* methods (such as hidden Markov models, Bayesian mixture models (Frühwirth-Schnatter, 2006; Todorov et al., 2020), and advanced clustering methods (Andreopoulos et al., 2009; Gerber and Horenko, 2015; Rodrigues et al., 2021)) ignore any prior assignments of data to labels and groups in a given dataset. Herewith, however, a lot of valuable information is lost.

Also in a broader context of unsupervised data anomaly detection, two major method families like the one-class support vector machines (OCSVM, sometimes also referred to as one-class learning methods) (Choi, 2009; Zhu et al., 2016) and isolation forests (IF, anomaly detection algorithms based on random forest ideas) (Liu et al., 2008; Hariri et al., 2021) rely either on the explicit knowledge of some subsets of correctly identified data anomalies that can be used for training or on knowing the exact proportion of anomalous data in the given dataset. In the latter case, providing the exact proportion of anomalous data in OCSVM and IF allows identifying the exact value of the anomaly threshold that can be used to separate normal data from anomalous data. The primary aim of this study is providing a robust computational mislabeling inference procedure for generalized linear models (e.g., logistic regressions)—an algorithmic procedure that does neither rely on the explicit knowledge of the particular mislabeled data instances nor on the knowledge of the exact proportion of the mislabeled data in the given dataset. Instead, the introduced procedure relies on the knowledge of the upper bound for the mislabeled data proportion and deploys the tools from information theory (like Akaike information criterion) to infer the optimal logistic model and the optimal class-specific mislabeling probability matrix. We thus address the currently existing methodological gap and propose a scalable method that can realistically be applied in the analysis of biomedical data. The method permits reduced sets of discriminative features to be inferred while co-estimating the group-specific data mislabeling risks. We apply the method to two examples: breast cancer diagnoses based on radiographs (example 1) and an analysis of genomic features from a Wellderly cohort (example 2) (Erikson et al., 2016)—a cohort of people who live to be 80 years or more without having experienced a serious or chronic disease. Analysis of a synthetic dataset (i.e., a dataset created by a generalized linear model with known parameters and known group-specific mislabeling, which mimics the breast cancer imaging data from example 1) is shown in Section 3 of the Supplementary Material.

## Materials and Methods

Here, we give a brief description of the methodology. Detailed mathematical derivation and an investigation of its mathematical properties (a case of mislabeled Bernoulli trials, proofs of conditions for existence and uniqueness of solutions, monotonicity, and convergence of the numerical method) can be found in Lemmas 1–5 in the Supplementary Material. We consider a problem of analyzing the labeled datasets $\left(X,Y^{\text{obs}}\right)$ that are grouped into *N*_*g*_ cohorts/groups, with *T*_*g*_ being the number of instances, for example, the number of patients in the cohort/group *g*.

For every data instance $\left(t,g\right)$ (for every patient number *t* in the group *g*), we would like to identify a relation between a vector of features *X*_*t*,*g*_ (an *n*-dimensional vector containing, e.g., the genotype, the age, and some other patient-specific information) and a “true”—but directly unobserved—categorical label *Y*_*t*,*g*_. This “true” label is taking values in the finite set of *m* categories *y* = {*y*_1_, *y*_2_, *…* , *y*_*m*_} and represents, for example, a certain phenotype. A typical setting would be to compare features from a cohort of ill people to a control group, resulting in *m* = 2 representing labels like *y*_1_ = “sick” and *y*_2_ = “healthy.” We consider the “true” labels *Y*_*t*,*g*_ to be unobserved since they are not directly available. Only the observed labelings $Y_{t,g}^{\text{obs}}$ are available and can be mislabeled in every instance $\left(t,g\right)$ with some, yet unknown, cohort-specific mislabeling probability $r_{i,j,g}=\left[Y_{t,g}=y_{i}|Y_{t,g}^{\text{obs}}=y_{j}\right]$ . We assume that a parametric (i.e., dependent on a vector of parameters *α*) discriminative model is establishing a conditional dependence between the particular “true” unobserved labeling and a particular observed feature vector *X*_*t*,*g*_. The parametric function relating features and labels is denoted as $\phi_{i}\left(X_{t,g},\alpha \right)$ . These parametric functions can, for example, be a generalized linear model (GLM, e.g., the standard logit and probit models for *m* = 2) (McFadden, 1974; Friedman et al., 2010) or a neuronal network (Hastie et al., 2009).

Let $y_{t,g,j}^{\text{obs}}=\chi \left(Y_{t,g}^{\text{obs}}=y_{j}\right)$ , where *χ* is the indicator function, taking value 1 if its argument is true and 0 otherwise. Then it can be shown that the unknown optimal model parameters α* can be inferred together with the optimal mislabeling risk matrix r* by solving the following maximization problem (see Section 1 of SI for a step-by-step derivation):

$$\begin{array}{ccc} \left(\alpha^{*},r^{*}\right) & = & \underset{\alpha ,r}{\mathrm{argmax}}L\left(\alpha ,r\right),\\ L\left(\alpha ,r\right) & = & \overset{N_{g},T_{g}}{\underset{g,t=1}{\sum }}\frac{1}{N_{g}T_{g}}\log\left(\overset{m}{\underset{i,j=1}{\sum }}y_{t,g,j}^{\text{obs}}r_{i,j,g}\phi_{i}\left(X_{t,g},\alpha \right)\right), \end{array}$$ (1)

which is subject to the following constraints:

$$\begin{array}{ccc} \overset{m}{\underset{i=1}{\sum }}r_{i,j,g} & = & 1,\;\forall g,j, \end{array}$$ (2)

$$\begin{array}{ccc} 0\leq r_{i,j,g}^{-} & \leq & r_{i,j,g}\leq r_{i,j,g}^{+}<1,\;\forall g,i,j, \end{array}$$ (3)

$$\begin{array}{ccc} |\alpha {|}_{1} & = & \overset{n}{\underset{d=1}{\sum }}|\alpha_{d}|\leq C, \end{array}$$ (4)

where $\left[r_{i,j,g}^{-},r_{i,j,g}^{+}\right]$ are user-defined intervals for mislabeling risks (based, e.g., on some prior knowledge) and *C* is the a priori unknown constant that implicitly confines the number of non-zero components of the parameter vector *α*. The user-defined choice of matrices *r* ^+/−^ is not really arbitrary and should be done based on prior knowledge in such a way that the r-constraints (3) do not lead to an empty set.

It is straightforward to verify that the particular case of problem (1–4) with fixed *r* being an identity matrix is equivalent to the widely used Lasso (or *l*1 − ) regularization methods introduced by Tibshirani (1996), Bair and Tibshirani (2004), Friedman et al. (2010), Simon et al. (2011), and Taylor and Tibshirani (2015). In context of these Lasso-regularized methods, decreasing the constant *C* in (4) one reduces the number of non-zero elements in *α*, thereby reducing the number of non-zero parameters and avoiding overfitting. This becomes especially important in biomedical applications, where the number *n* of model parameters is large compared with the size of the available statistics—a typical scenario when the danger of overfitting becomes imminent.

**FIGURE 1:**
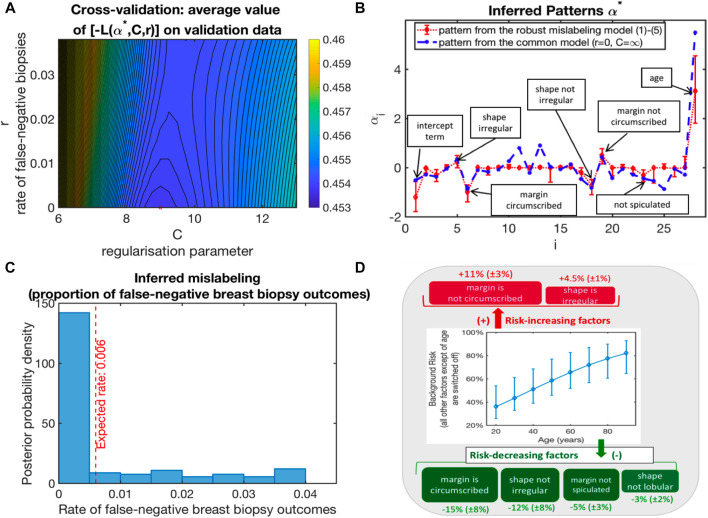
Application of (1–4) to the analysis of the standard BI-RADS dataset from http://archive.ics.uci.edu/ml/datasets/Mammographic+Mass: **(A)** Model selection by cross-validation (bootstrap-averaged values of the functional **L** from (1) with optimal parameters from the training sets being evaluated on the validation datasets); **(B)** optimal parameter vector α*; **(C)** probability of a malignant diagnosis as a function of BI-RADS features for the two groups of patients; **(D)** average impact of single BI-RADS features (sensitivity of the risk to the 7 binary features of importance).

**FIGURE 2:**
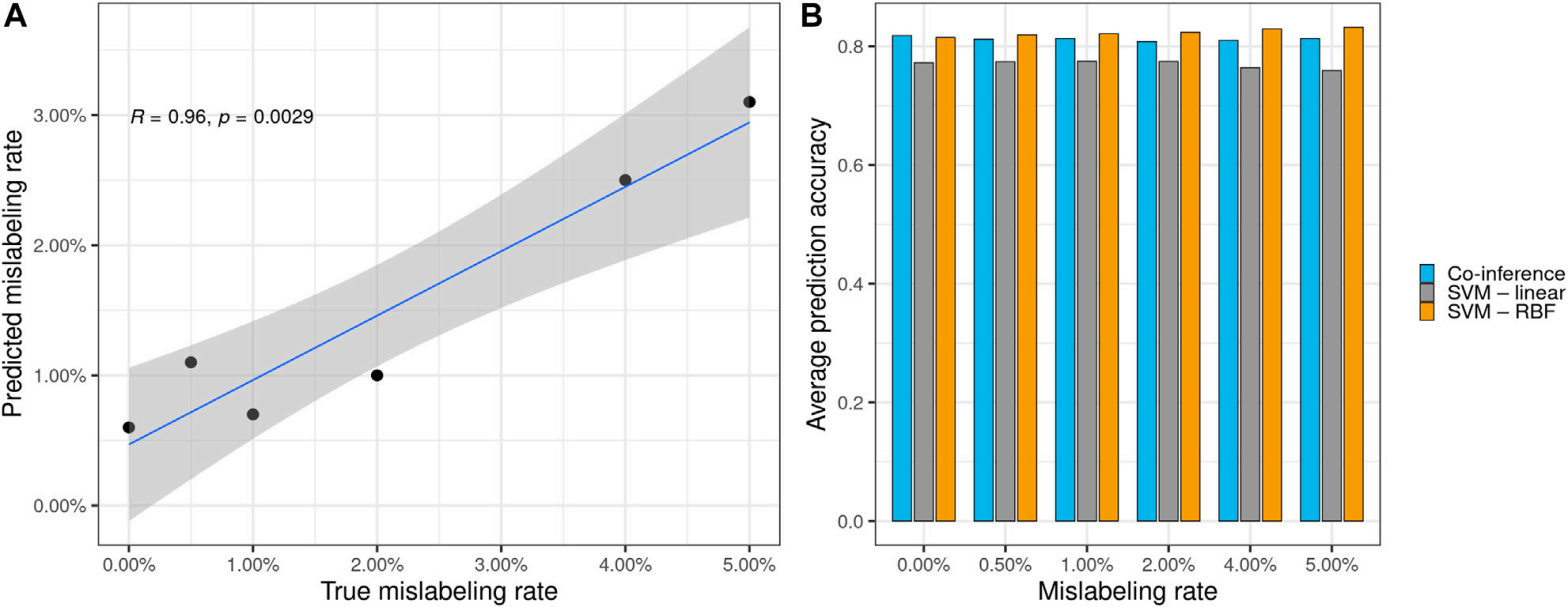
**(A)** Significant correlation between the true synthetically induced mislabeling rate of the mammography data and the error rate predicted by the co-inference method. **(B)** Performance of different model types on the mammography dataset with various mislabeling rates. All models, except the original one, were trained using the respective mislabeled dataset. The average prediction accuracy was calculated based on the original mammography dataset. Co-inference outperforms a linear SVC and performs nearly on par with a state-of-the-art SVC using an RBF kernel.

As proven in Section 2 of the SI, given the imperfectly labeled datasets $\left(X,Y^{\text{obs}}\right)$ , the solution of this optimization problem (1–4) results in the parameter vector α* for any fixed combination of *r* and *C*. This solution will be optimal in the case of the log-likelihood, that is, choosing this particular α* will result in a maximal probability for observing the given data $\left(X,Y^{\text{obs}}\right)$ , confined to a particular choice of *r* and *C*. Selection of the optimal parametric model class *ϕ*, as well as selection of parameters *r* and *C*, can be approached with the standard model selection procedures of machine learning, for example, by means of the cross-validation, with the help of the information criteria or through selective inference (Burnham and Anderson, 2002; Zhang et al., 2010; Taylor and Tibshirani, 2015) (see, e.g., Figure 1A). Uncertainty of the obtained mislabeling risks r* and model parameters α* can be obtained using the common non-parametric bootstrap sampling procedure (Efron and Tibshirani, 1993) (see, e.g., Figures 1C,E, 2A).

If *m* and *N*_*g*_ are not too large, one can use the following numerical scheme: 1) first, the rectangular domain spanning a set of admissible values for mislabeling matrix elements in (3) and admissible values of *C* in (4) is sampled (e.g., by means of a uniform equidistant grid), and 2) for every particular grid point (*r*_*s*_, *C*_*s*_), one deploys some standard gradient-based optimization method to solve (1–4). In the second step (ii), one can use an interior-point method or the sequential quadratic programming (Nocedal and Wright, 2006)), performing constrained concave optimization of (2) subject to a constraint (4) only, with fixed values of *r*_*s*_ and *C*_*s*_. For example, when *m* = 2 and *N*_*g*_ = 1 (the case emerging in examples 1 and 2), there will be only two independent parameters in *r*. Together with the scalar dimension for the regularization constant *C*, this will result in a 3D grid (*r*_*s*_, *C*_*s*_).

With respect to the model function *ϕ*, optimization of the concave problem (1–4) would only require the evaluation and communication of the function values and the gradients of *ϕ* with respect to *α*. This means that a solution of the overall problem (1–4) can be easily integrated into the common software packages for labeled data analysis. Moreover, solutions of problem (1–4) for different particular choices of (*r*_*s*_, *C*_*s*_) can be found completely independent of each other, herewith allowing for a highly scalable (“embarrassingly parallel”) implementation on high-performance computing facilities.

## Results

### Example 1: Breast cancer diagnostics based on the standard BI-RADS X-ray imaging data.

First, we consider an analysis and feature selection problem for the standard breast cancer BI-RADS dataset based on X-ray imaging. This dataset is available for open access at the UCI Machine Learning Repository http://archive.ics.uci.edu/ml/datasets/Mammographic+Mass and contains information from biopsies of 403 healthy (benign) subjects and 427 malignant breast cancer patients. For each patient, six attributes are given: 1) the BI-RADS assessment (with values from 1 to 5), 2) the patient’s age, 3) the mass shape (with 4 subcategories), 4) the mass margin (with 5 subcategories), 5) the mass density (with 4 subcategories), and 6) the final binominal label for severity: benign = 0 or malignant = 1. This results in a set of 26 binary features for the shape, margin, and density and one real-valued age feature, that is, in total, we have 27 features to consider. This standard categorical dataset is widely used to access the quality of various computer-aided diagnostic tools (CADs), with the general aim of identifying such a CAD that would use non-invasive information of age and mammographic image features for the precise diagnostics of breast cancer (Elder et al., 2007; Ayer et al., 2010; Zhang et al., 2010).

The standard measure for CAD performance adopted in the medical literature is called area under curve (AUC) (Qin and Hotilovac, 2008). The closer the AUC value is to 1.0, the better is the performance of the respective CAD and the lower is the probability of a false-positive or false-negative diagnosis. To compute the AUC values of different CADs—together with 95% confidence intervals of AUC, we use the methodology described in Qin and Hotilovac (2008). The implementation of this method is available for open access at https://github.com/brian-lau/MatlabAUC.

The given label “benign” or “malignant” (*m* = 2) in the dataset is obtained based on an invasive core-needle biopsy analysis of the tissue. Whereas the rate of the false-positive core-needle breast biopsy outcomes is practically zero, the rate of the false-negative biopsy findings can be quite significant. According to the literature, it can vary in a wide range between 0.005 and 0.19 (Shah et al., 2003; Verkooijen et al., 2004; Boba et al., 2011). CADs based on artificial neuronal networks (ANNs) have been reported to have the highest AUC for these data (Elder et al., 2007; Ayer et al., 2010; Gerber and Horenko, 2017). Training such ANNs results in an AUC of 0.85 with an 95% confidence interval of $\left[0.82,0.88\right]$ , whereas using the standard BI-RADS diagnostics (on the same data and computed deploying the methodology from Qin and Hotilovac (2008), one obtains an AUC of 0.82 with a 95% confidence interval of $\left[0.78,0.84\right]$ .

However, published CAD methodologies that used this standard BI-RADS dataset for training do not consider an eventual risk of mislabeling due to wrong biopsy outcomes. As mentioned before, this risk may achieve 0.19 (Shah et al., 2003; Verkooijen et al., 2004; Boba et al., 2011). If there are mislabelings in the data, then the AUC of the validation set is biased and misleading.

To apply the suggested methodology presented here, we set the broad a priori bounds for mislabeling risks (with *r* ^−^ = 0, *r* ^+^ = 0.5) and tested various model classes *ϕ*, including the naive Bayesian classifier models, linear models, probit models, and logit models. The optimal results are achieved for *ϕ* being a logit model function. Analysis results are summarized in Figure 1.

Figure 1A shows the negative values of *L* from optimal (1) with *α*∗ obtained for the training sets and evaluated on the validation sets, averaged over 500 random cross-validations, when the full dataset is randomly divided into the training set (with 75% of the data) and the validation set (25% of the data). It appears that the cross-validation optimal model is characterized by the false-negative biopsy rate *r*∗ close to zero and the optimal regularization parameter value C around eight. Figure 1B visualizes the optimal parameter vector *α*∗ with the features of interest together with their impact.

Furthermore, to estimate the confidence intervals for *α*∗ and *r*∗, we deploy the common non-parametric bootstrap sampling procedure (Efron and Tibshirani, 1993) (see Figure 1C). The resulting posterior distribution of inferred false-negative biopsy outcomes is shown in Figure 1C, together with their expected posterior estimate—being 0.6*%*. As can be seen from Figures 1C,D, the obtained diagnostic model is robust and contains the binary yes/no characteristics from just three features that statistically significantly influence malignancy risk (from age, margin, and shape of the inclusion). In contrast, commonly applied diagnostic strategies include 28 characteristics from four features, including an additional intrusion density feature (with 4 categories). Age appears to be the most significant feature, almost doubling the average risk for individuals older than 80 years as compared to twenty-year-olds. Besides age, there are two binary factors that increase malignancy risk (“margin is not circumscribed” and “shape is irregular”) and four risk-decreasing binary factors (“margin is circumscribed,” “shape is not irregular,” “margin is not spiculated,” and “shape is not lobular”).

In a perfect situation, one would need to have a validation set with 100% correct labelings to make the comparison of different diagnostic strategies with respect to their AUC. Since such datasets are not available in the published medical literature, we follow a different way and investigate and compare the systematic bias that is imposed by the latent mislabeling risks on the AUC values of different diagnostic procedures. In order to better address the aforementioned issue of AUC with mislabelings to the bias induced by different mislabeling rates, we computed and compared the bootstrap confidence intervals of the systematic bias that is introduced by the latent mislabeling risks on the AUC values of different diagnostic procedures (see Supplementary Figure S2 from the Supplement).

The application of the introduced strategy described in the *Methods* section to these BI-RADS data revealed that it is almost perfectly labeled (inferred expected malignant mislabeling rate is almost 0% and the benign mislabeling rate is 0.6%). This result is in agreement with the range of false-negative biopsy outcomes from the clinical reports (Shah et al., 2003; Verkooijen et al., 2004; Boba et al., 2011). The AUC values obtained with the mislabeling model (1–4) introduced in the article are statistically significantly higher than the AUC values of the BI-RADS and of the ANN strategies (without considering potential mislabeling) published in the literature. These results indicate that the obtained classification model with mislabeling would have AUC values that are statistically significantly higher than the common BI-RADS diagnostics.

Furthermore, the logit model performs robustly, despite the number of mislabelings, and offers a good estimate with respect to the mislabeled data, as indicated by the calculated the Pearson correlation between the estimated and true mislabeling rates (R = 0.096, *p* = 0.002 9) (see Figure 2A). Additionally, we evaluated our logit model against two types of support vector classifiers (SVCs) with linear and radial basis function (RBF) kernels to compare model performance apart from the aforementioned AUC metric. For this, we trained all models with 200 bootstrap steps on synthetically mislabeled datasets based on the breast cancer data with known mislabeling rates and calculated their average model accuracy with respect to the original breast cancer data. The logit model outperforms the linear SVC and performs nearly on par with a state-of-the-art SVM with RBF kernel (see Figure 2B and Supplementary Material Chapter S3, Supplementary Table S1). Compared to the SVM with RBF kernel, which performs about equally well, our method is the only one that can predict the expected mislabeling rate. This prediction—although generally somewhat less than the true value—shows a strong correlation between the true synthetically induced mislabeling rate of the mammography data and the error rate predicted by the co-inference method (see Figure 2A).

### Example 2: Wellderly Data Analysis and Extraction of Genomic Patterns of Healthy Aging

In the second example, our method is applied to human sequencing data in the context of genome-wide association studies (GWASs), where a population sharing a trait is compared to a “normal” control population. The sample consists of the Wellderly cohort, a cohort of healthy elderly (older than 80 years) individuals (Erikson et al., 2016), and the Caucasian population of phase 3 from the “1000 Genomes Project” (1 KG), which serves as the control (The 1000 Genomes Project Consortium, 2015). The analytical question would be to compare both groups and to find genetic patterns that correlate with the “Wellderly” phenotype.

GWAS may suffer a number of statistical errors, such as overfitting, *p*-value misinterpretation, or batch effects due to different sequencing platforms (Nuzzo, 2014; Gerber et al., 2020; Pfenninger et al., 2021; Weißbach et al., 2021). In our Wellderly control cohort, we face the additional problem of almost certain sample mislabeling between “cases” and controls, which is impossible to prevent. Specifically, some of the individuals from the control group may actually progress to be healthy individuals of advanced age and thus belong to the Wellderly population and not the control population. It would be impractical to wait several decades to observe the outcome.

For the analysis, precomputed VCF files were obtained from the 1000 Genomes Project and the Wellderly cohort. Then the cohorts were merged and filtered (minimum allele frequency filter of 1%, no missing genotypes, only SNPs that appear in both cohorts). The merged cohort was subset further to 163 individuals from each population, who were most closely related (according to the genomic distance) and whose self-reported Caucasian inheritance was above 95% in the Wellderly cohort, to counteract population stratification. Lastly, the vcf data were filtered to only contain biallelic SNPs, since the model does not yet accommodate other SNP types, and the vcf entries were recoded as 0, 1, and 2 to indicate major minor or mixed alleles. The final cohort was split into training and test data (25 and 75%, respectively) and the mathematical model introduced above (1–4). A non-parametric bootstrap sampling paradigm was used for independent random separations of the cohort into training and validation groups. All 100 results from applying (1–4) to each of these random training and validation choices were used to create the posterior probability density functions of mislabeling risks for the two data groups, as well as to compute the 95% confidence intervals for feature weights and for individual Wellderly probabilities in the groups. Optimal results appear to be achieved with the logit model function *ϕ*.

**FIGURE 3:**
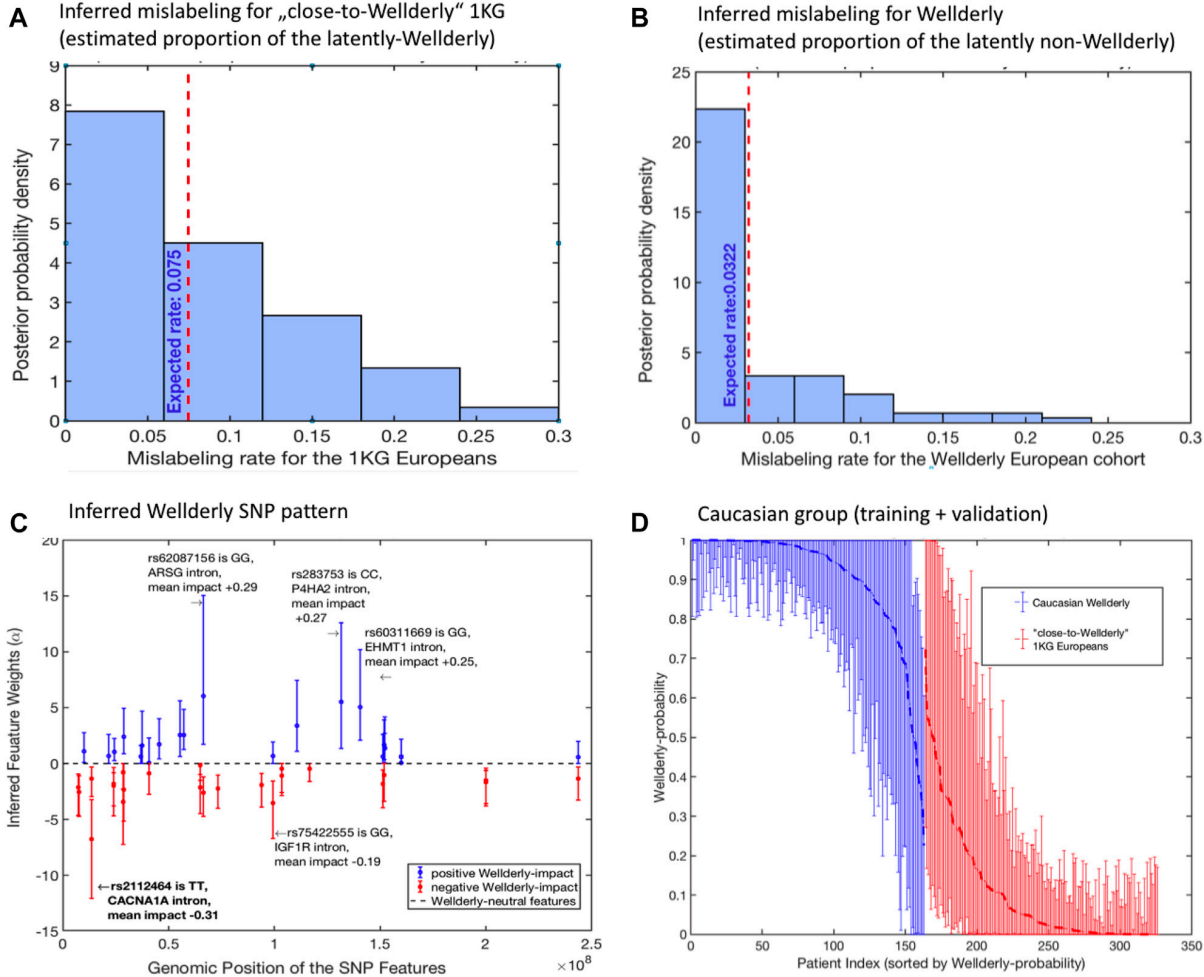
Application of (1–4) to the analysis of filtered SNP data from (Erikson et al., 2016): **(A)** posterior probability distribution of the inferred optimal mislabelings from the “close-to-Wellderly European” cohort from 1000 Genomes (basis for the control group); **(B)** posterior probability distribution of the inferred optimal mislabelings from the “Wellderly Caucasian” cohort (Erikson et al., 2016) (basis for the case group); **(C)** estimated optimal weights *α*_*i*_ of the SNP patterns together with their 95% confidence intervals; **(D)** individual probabilities of Wellderly being due to genomic factors (together with their 95% confidence intervals), as inferred from the optimal feature weights *α* from panel **(C)**. Posterior distributions in **(A,B)** and confidence intervals in **(C,D)** are obtained by means of the non-parametric bootstrap sampling (Efron and Tibshirani, 1993) with 100 ensemble realizations.

First, the mislabeling probability between Wellderly and control individuals was assessed. Here, the estimated posterior probability for the perfect group labeling altogether was only 0.09, meaning that samples were mislabeled between Wellderly and control with a probability of 91*%*. Figures 3A,B illustrate the expected proportion of “mislabeled” individuals in each cohort. According to the model, there were 7.5% control cases mistakenly classified as Wellderly and about 3.2% Wellderly individuals possibly mislabeled.

Then the feature weights *α* were recorded, which means that the SNPs were statistically relevant to the Wellderly phenotype, according to the model. Forty-five SNP features had statistically significant values of *α*. The full list of features, together with their estimated mean impacts on Wellderly probability, is provided in Section 3 of the SI. Note that this result does not imply that only these 45 features have a significant impact on the healthy ageing probability, and it does not mean that the other features (that got zero weights *α*_*i*_) are completely unrelated to the Wellderly genotype. As mentioned before, deploying our model (1–4), one can identify a unique compact pattern of features. However, the model tries to establish a consensus between finding the largest number of informative features and overfitting, which may result in some SNPs not labeled as informative simply because the model tries to correct for potential overfitting, and vice versa.

The investigation of these 45 SNP features revealed rs429358, situated in the coding region of the APOE gene. rs429358 is one of two markers that define the APOE-E4 status, which is the strongest common genetic risk factor for Alzheimer’s disease. The remainder of statistically significant SNP features are either intronic (38 SNPs) or intergenic (6 SNPs), where intergenic SNPs were often near a gene bearing a statistically significant intronic SNP. In fact, the 45 SNP features tended to cluster within a smaller number of genes 28 genes (see the table in Supplementary Material), suggesting multiple independent genetic signals are present within these genes. Further inspection of these genes reveals dramatic enrichment of genes associated with longevity, including lipid metabolism genes: APOE, APOC3, and CETP; insulin signaling and mTOR signaling: ADCY2, AKT3, CREB5, IGF1R, INSR, PIK3CD, and RHEB; and AMPK-dependent metabolic signaling: CAMK4, PPARGC1A, PRKAA1, and PRKAG2.

## Discussion

Although the raw datasets used in many types of biomedical data analysis are very large, making sense of them requires statistical methods able to handle data problems where the dimensionality *n* of their feature spaces is typically orders of magnitude larger than the number *T* of individuals in the groups. According to central limit theorems, the uncertainty of typical parameter estimation procedures will reduce as *T* ^1/2^ with the growing statistics size *T* only if the individual data instances in the statistics can be assumed to be independent. The more that dependence between the data instances, the slower will be the rate of this uncertainty reduction with *T*. In this respect, genomic SNP data pose special problems as they contain a huge fraction of dependencies between SNP pairs that are in a linkage equilibrium with each other. Another source of bias is introduced through the impact of latent/unobserved factors (e.g., in the form of stratification effects and latent variables) and various forms of mislabeling. Mislabeling can bias the results obtained by standard relation measures that dwell on an exact labeling assumption (e.g., it can bias the results of the *t*-test, chi-square test, Fisher’s exact test, odds ratio, and many other methods). Common methods to overcome this problem, including outlier detection methods and HMMs, either rely on the presence of some subsets that are exactly labeled or introduce additional variables that estimate the probability of every particular data point being an outlier. This significantly increases the dimension of the parameter space and the risk of overfitting. These problems remain an issue even when the numbers of individuals sequenced by platforms such as *23andme* approach millions. All these issues (*n* > > *T*, violation of the independence assumption, latent impacts, and mislabeling) lead to uncertainty in estimating parameters and make biomedical GWAS applications very challenging. These issues also limit the applicability of advanced Big Data tools such as artificial neuronal networks to problems of this type. A promising direction toward solving these problems can be found in methods based on Lasso regularization ideas first introduced by R. Tibshirani and coworkers (Tibshirani, 1996; Bair and Tibshirani, 2004; Friedman et al., 2010; Simon et al., 2011; Taylor and Tibshirani, 2015), through a robust and computationally efficient shrinkage of the feature space and zeroing out of less relevant feature components. As demonstrated before, introducing a measurement of the probability of group-specific mislabeling and deploying Bayesian tools permit a natural extension of these ideas to situations in which none of the data groups is presumed perfectly labeled. This is accomplished without introducing many new parameters that have to be estimated. For example, in the case of a one-data group (*N*_*g*_ = 1) with two data labels (*m* = 2, e.g., “Wellderly” and “non-Wellderly” labels in example 2), only two additional mislabeling parameters need to be estimated. The open-source MATLAB implementation we provide here permits implementation of the algorithm in a strongly scalable way. A full analysis of the data in example 2 takes 24 days on a single-core PC, 2 days on a PC workstation with 12 cores, and only 5 h on a small-scale computer cluster with hundred nodes. Measuring the performance of such methods has a general problem due to the presence of latent impacts and mislabeling, which can bias either standard measures such as AUC, accuracy scores, and Fisher’s exact test or and linkage disequilibrium measures. In future studies, a better understanding of ever-growing sets of biomedical data requires the further development of robust and computationally scalable relation measures that can explicitly infer and take into account eventual latent effects and mislabeling.

## Data Availability

Publicly available datasets were analyzed in this study. These data can be found in the following site; a parallel MATLAB implementation of this method is provided for open access via GitHub: https://github.com/SusanneGerber/Mislabeling_Coinference/tree/master/Release/Mislabeling_Coinference. The breast cancer BI-RADS dataset is available for open access at the UCI Machine Learning Repository: http://archive.ics.uci.edu/ml/datasets/Mammographic+Mass. Aggregate unfiltered annotated variants for healthy-ageing Caucasian individuals (Wellderly) and their allele and genotype frequencies are available via Scripps Translational Science Institute Variant Browser: https://genomics.scripps.edu/browser.
