# Supplementary material for: Co-Inference of Data Mislabelings Reveals Improved Models in Genomics and Breast Cancer Diagnostics
Source: Front Artif Intell. 2022 Jan 5;4:739432. doi: 10.3389/frai.2021.739432 (PMC8766632; doi:10.3389/frai.2021.739432)
Supplement: Supplementary file 1 [file DataSheet1.PDF]

Supplementary Material

This supplement is subdivided into the four following chapters:

1. Derivation of the optimized formulation for the data driven model inference problem with latent mislabeling risks.
2. Mathematical properties of the problem, numerical methods.
3. Performance on synthetically mislabeled mammography data
4. List of SNPs relevant for Caucasian Wellderly

1 DERIVATION OF THE OPTIMIZING FORMULATION FOR THE DATA DRIVEN MODEL INFERENCE PROBLEM WITH LATENT MISLABELING RISKS

Variables and notation:

We will consider a problem of analysing the labeled data sets $\left(X, Y^{\text{obs}}\right)$ that are grouped into N_g cohorths/groups, with T_g being the number of instances (e.g., the number of patients) in the cohort/group g. For every data instance (t, g) (e.g., for every patient number t in the group g), we would like to identify an optimal robust relation between a vector of features $X_{t,g}$ (e.g., an n-dimensional vector containing the genotype, the age, some other patient-specific informations) and a "true" - but directly unobserved - categorical labels $Y_{t,g}$. $Y_{t,g}$ is taking values in the finite set of m categories $y = \{y_1, y_2, \ldots, y_m\}$, representing for example a certain phenotype, e.g., with $m = 2$ for labels like y_1 ="sick" and y_2="healthy". We consider the "true" labels $Y_{t,g}$ to be unobserved since they are not available directly. Directly given are only the observed labelings $Y^{\text{obs}}_{t,g}$, that can be mislabeled in every instance (t, g) with some - yet unknown - probability $r_{i,j,t,g} = \left[Y_{t,g} = y_i | Y^{\text{obs}}_{t,g} = y_j\right]$. As $\phi_i\left(X_{t,g}, \alpha\right) = \left[X_{t,g} | Y_{t,g} = y_i, \alpha\right]$ we will define a parametric stochastic model (i.e., a probabilistic model dependent on the finite-dimensional model parameter vector α) that establishes a conditional dependence between the particular labeling and the probability of a certain feature vector realisation. This model should be chosen such that it satisfies:

$$\sum_{i=1}^{m} \phi_i\left(X_{t,g}, \alpha\right) \equiv 1, \quad \forall t, g, \alpha. \tag{S1}$$

Such parametric stochastic models can for example be the generalised linear models (GLMs, e.g., the standard logit and probit models for $m = 2$) [7, 3] or the neural networks [5]. We will assume that the model function class ϕ is pre-defined and fixed - and that the parameter vector α should be inferred in the data analysis procedure together with the unknown mislabeling probabilities r. Selection of the optimal parametric model class ϕ can be approached with the standard model selection procedures from the machine learning (e.g., by means of the cross-validation or with the help of the information criteria) [2].

Derivation of the nonparametric and nonhomogenous log-likelihood formulation:

Deploying the notation introduced above and getting use of the *law of the total probability* [5], we can write the probability of observing a certain feature vector in the particular data instance (t, g) as:

$$\left[X_{t,g}\right] = \sum_{i=1}^{m} \left[X_{t,g} | Y_{t,g} = y_i\right] \left[Y_{t,g} = y_i\right]. \tag{S2}$$

Next, we use the *law of the total probability* again, establishing a relation between the "true" labeling Y and the observed labeling Y^{obs}:

$$[Y_{t,g} = y_i] \quad = \quad \sum_{j=1}^{m} \left[Y_{t,g} = y_i | Y_{t,g}^{\text{obs}} = y_j\right] \left[Y_{t,g}^{\text{obs}} = y_j\right]. \tag{S3}$$

Incerting (S3) into (S2) and assuming the statistical independence of pairs $(X_{t_1,g_1}, Y_{t_1,g_1}^{\text{obs}})$ and $(X_{t_2,g_2}, Y_{t_2,g_2}^{\text{obs}})$ for $(t_1, g_1) \neq (t_2, g_2)$, we can express the overall probability (called *likelihood*) for observing the given data as:

$$\left[X, Y^{\text{obs}}\right] \quad = \quad \prod_{g=1}^{N_g} \prod_{t=1}^{T_g} \sum_{i,j=1}^{m} [X_{t,g} | Y_{t,g} = y_i] \left[Y_{t,g} = y_i | Y_{t,g}^{\text{obs}} = y_j\right] \chi\left(Y_{t,g}^{\text{obs}} = y_j\right), \tag{S4}$$

where $\chi(x)$ is an indicator function taking the value one if its argument is true and value zero otherwise. Then, given the data sets $\left(X, Y^{\text{obs}}\right)$, we can infer both the model relations $[X_{t,g}|Y_{t,g} = y_i]$ and an $m \times m$ matrix of mislabeling risks $\left[Y_{t,g} = y_i | Y_{t,g}^{\text{obs}} = y_j\right]$ by maximising the following log-likelihood function:

$$\log\left[X, Y^{\text{obs}}\right] \quad = \quad \sum_{g=1}^{N_g} \sum_{t=1}^{T_g} \log \sum_{i,j=1}^{m} [X_{t,g} | Y_{t,g} = y_i] \left[Y_{t,g} = y_i | Y_{t,g}^{\text{obs}} = y_j\right] \chi\left(Y_{t,g}^{\text{obs}} = y_j\right). \tag{S5}$$

In order to preserve the probability properties of the optimisation arguments $[X_{t,g}|Y_{t,g} = y_i]$ and $\left[Y_{t,g} = y_i | Y_{t,g}^{\text{obs}} = y_j\right]$, the log-likelihood maximisation should be subject to the equality and inequality constraints:

$$\begin{aligned} [X_{t,g}|Y_{t,g} = y_i] &\geq 0, \quad \forall t, g, i, \\ \left[Y_{t,g} = y_i | Y_{t,g}^{\text{obs}} = y_j\right] &\geq 0, \quad \forall t, g, i, j, \\ \sum_{i=1}^{m} \left[Y_{t,g} = y_i | Y_{t,g}^{\text{obs}} = y_j\right] &= 1, \quad \forall t, g, j. \end{aligned} \tag{S6}$$

However, it is easy to verify that the overall number of the unknown optimisation arguments (the values of $[X_{t,g}|Y_{t,g} = y_i]$ and $\left[Y_{t,g} = y_i | Y_{t,g}^{\text{obs}} = y_j\right]$ for different t, g, i and for different realisations of X) is much larger then the overall size of the available data $\left(X, Y^{\text{obs}}\right)$ - easily resulting in an effect that is known as *overfitting*. This means that the optimisation problem (S5-S6) is underdetermined and would provide multiple solutions that depend on the initialisation/starting point of the numerical optimisation procedure. This general problem feature is known as *ill-posedness* [4], a remedy for this problem can be provided by a procedure called *regularisation* and will be explained in the next paragraph.

Making the problem formulation well-posed, additional regularisation assumptions:

To make the problem formulation (S5-S6) *well-posed*, we will deploy two additional assumptions. As demonstrated in the Examples part of the main manuscript, omitting one of these assumptions can easily result in the ill-posed and overfitted models.

Assumption 1: mislabeling risks $\left[Y_{t,g}=y_i|Y_{t,g}^{\text{obs}}=y_j\right]$ are cohort/group-specific, do not depend on t, i.e.:

$$r_{i,j,g} \equiv \left[Y_{t,g}=y_i|Y_{t,g}^{\text{obs}}=y_j\right], \quad \forall t \tag{S7}$$

and for all i,j,g are confined to some a priori defined mislabeling risk intervals $\left[r_{i,j,g}^{-}, r_{i,j,g}^{+}\right]$, i.e.:

$$0 \leq r_{i,j,g}^{-} \leq r_{i,j,g} \leq r_{i,j,g}^{+} < 1, \quad \forall g,i,j. \tag{S8}$$

Assumption 2: there exists an explicit set of functions $\phi_1, \phi_2, \ldots, \phi_m$ describing a relation between the m categories of the "true" unobserved labeling variables Y and the feature vectors X:

$$\phi_i\left(X_{t,g},\alpha\right) \equiv \left[X_{t,g}|Y_{t,g}=y_i\right], \quad \forall t,g, \tag{S9}$$

such that the condition (S1) is fulfilled and that the set of parameters α is *regular* in $l1$-sense [11], i.e.:

$$|\alpha|_1 \leq C. \tag{S10}$$

Assumption 1 is reasonable since in many biomedical applications the probability of data mislabeling is mostly independent of t and X_t - and the data can be grouped into N_g cohorts in such a way that the a priori available knowledge about the lower and upper bounds $r_{i,j,g}^{-}$ and $r_{i,j,g}^{-}$ for mislabeling risks can be used in each of the resulting cohorts. Illustrations of this approach - that gets use of such a prior knowledge about $r_{i,j,g}^{-}$ and $r_{i,j,g}^{-}$ - can be found in both application examples from the main manuscript.

Assumption 2 is known under the name *lasso-regularisation* [11, 3, 10]. In context of generalised linear models it has been demonstrated to be one of the most efficient tools against model overfitting [5]. On a practical side, imposing constraint (S10) for decreasing values C results in zeroing-out the unsignificant elements of the model parameter vector α and allows finding a minimal sufficient set of non-zero model parameters [3].

Substituting (S1,S7,S8,S9,S10) into (S5-S6) and dividing by constants N_g, T_g we get the following constrained maximisation problem:

$$L\left(\alpha,C,r\right) = \frac{1}{N_g}\sum_{g=1}^{N_g}\frac{1}{T_g}\sum_{t=1}^{T_g}\log\left(\sum_{i,j=1}^{m}\phi_i\left(X_{t,g},\alpha\right)r_{i,j,g}\chi\left(Y_{t,g}^{\text{obs}}=y_j\right)\right) \to \max_{\alpha,r}, \tag{S11}$$

$$\sum_{i=1}^{m}\phi_i\left(X_{t,g},\alpha\right) \equiv 1, \quad \forall t,g,\alpha, \tag{S12}$$

$$\phi_i\left(X_{t,g},\alpha\right) \geq 0, \quad \forall t,g,i, \tag{S13}$$

$$\sum_{i=1}^{m} r_{i,j,g} = 1, \quad \forall g,j, \tag{S14}$$

$$0 \leq r_{i,j,g}^{-} \leq r_{i,j,g} \leq r_{i,j,g}^{+} < 1, \quad \forall g,i,j, \tag{S15}$$

$$|\alpha|_1 \leq C. \tag{S16}$$

Most of the standard probabilistic model functions ϕ_i (e.g., logit and probit functions, other standard transfer functions in neuronal networks) fulfil the constraints (S12) and (S13) automatically. In such cases these two constraints can be omitted, resulting in the following optimisation problem formulation:

$$L(\alpha, C, r) = \frac{1}{N_g}\sum_{g=1}^{N_g}\frac{1}{T_g}\sum_{t=1}^{T_g}\log\left(\sum_{i,j=1}^{m}\phi_i\left(X_{t,g},\alpha\right)r_{i,j,g}\chi\left(Y_{t,g}^{\text{obs}}=y_j\right)\right)\to\max_{\alpha,r}, \quad \text{(S17)}$$

$$\sum_{i=1}^{m} r_{i,j,g} = 1, \quad \forall g,j, \quad \text{(S18)}$$

$$0 \leq r^{-}_{i,j,g} \leq r_{i,j,g} \leq r^{+}_{i,j,g} < 1, \quad \forall g,i,j, \quad \text{(S19)}$$

$$|\alpha|_1 \leq C. \quad \text{(S20)}$$

Set determined by the constraints (S18),(S19) is user-defined and non-empty - since $m > 1$ (and because categorical processes with only one state/category (m=1) make little sense). Moreover, a user-defined choice of $r^{+/-}$ is not really arbitrary and should be done in such a way that the r-constraints do not lead to an empty set.

It is straightforward to verify that for every t, g in the data there exists a j such that the respective indicator function $\chi\left(Y_{t,g}^{\text{obs}}=y_j\right)=1$. In other words, every data point in every cohort is assumed to be labeled. If the model function ϕ is strictly bigger then zero for all arguments on the open interval $(-\infty,+\infty)$ then the argument of the logarithm is always non-zero, the log-likehood function (S17) can not attain the value $-\infty$ and will be smoothly differentiable (if the model function ϕ is smoothly differentiable). These conditions are fulfilled for all of the common generalized linear models (e.g., for logit and probit models).

Even through the logarithm is a non-linear function, in the case of arbitrary characteristic binary coefficients $\chi_j \in \{0,1\}$, $\sum\limits_{j=1}^{m}\chi_j = 1$ of linear combination of general values $v_j \in \mathbb{R}$, we are able to write

$$\log\sum_{j=1}^{m}\chi_j v_j = \sum_{j=1}^{m}\chi_j\log v_j,$$

and therefore the terms in objective function (S18) with particular indexes t, g can be written in form

$$\begin{aligned}\log\left(\sum_{i,j=1}^{m}\phi_i\left(X_{t,g},\alpha\right)r_{i,j,g}\chi\left(Y_{t,g}^{\text{obs}}=y_j\right)\right) &= \log\left[\sum_{j=1}^{m}\left(\chi\left(Y_{t,g}^{\text{obs}}=y_j\right)\sum_{i=1}^{m}\phi_i\left(X_{t,g},\alpha\right)r_{i,j,g}\right)\right]\\ &= \sum_{j=1}^{m}\chi\left(Y_{t,g}^{\text{obs}}=y_j\right)\log\left(\sum_{i=1}^{m}\phi_i\left(X_{t,g},\alpha\right)r_{i,j,g}\right).\end{aligned} \quad \text{(S21)}$$

To simplify the analysis of the objective function (S17), let us denote $\hat{j}_{t,g} \in \{1,\ldots,m\}$ as index for which $\chi\left(Y_{t,g}^{\text{obs}}=y_{\hat{j}_{t,g}}\right)=1$. For every t, g there always exists exactly one $\hat{j}_{t,g}$, for all other $j \in \{1,\ldots,m\}\setminus\{\hat{j}_{t,g}\}$ in given term of t, j is the value of the indicator function equal to zero. Inserting (S21) into the objective function (S17) we obtain

$$L(\alpha, C, r) = \frac{1}{N_g}\sum_{g=1}^{N_g}\frac{1}{T_g}\sum_{t=1}^{T_g}\log\left(\sum_{i=1}^{m}\phi_i\left(X_{t,g},\alpha\right)r_{i,\hat{j}_{t,g},g}\right). \quad \text{(S22)}$$

2 MATHEMATICAL PROPERTIES OF THE PROBLEM, NUMERICS

Existence of a solution:

We suppose that

$$\forall r, g, i, j : \phi_i \left(X_{t,g}, \alpha \right) > 0, \quad r_{i,j,g} \geq 0,$$

which ensures that the arguments of logarithms in (S17) are positive, therefore the objective function is well defined on feasible set. If functions ϕ_i are continuous, then the whole objective function is continuous. Moreover, the feasible set is closed and convex, therefore by Weierstrass extreme value theorem [9], the problem has always a solution.

Bernoulli trials with mislabeling:

For the sake of simplicity let us consider the case with one cohort $N_g = 1$, two categories $m = 2$, and a constant parametric stochastic model, i.e., with

$$\forall t, g : \quad \phi_1 \left(X_{t,g}, \alpha \right) =: \alpha, \quad \phi_2 \left(X_{t,g}, \alpha \right) =: 1 - \alpha. \tag{S23}$$

Such a model satisfies condition (S12) and depends only on one t-independent unknown parameter $\alpha \in [0, 1]$. The remaining unknowns of the problem consist of mislabeling probabilities

$$r = \begin{bmatrix} [Y_{t,g} = y_1 | Y^{\text{obs}}_{t,g} = y_1] & [Y_{t,g} = y_1 | Y^{\text{obs}}_{t,g} = y_2] \\ [Y_{t,g} = y_2 | Y^{\text{obs}}_{t,g} = y_1] & [Y_{t,g} = y_2 | Y^{\text{obs}}_{t,g} = y_2] \end{bmatrix} = \begin{bmatrix} r_{1,1} & r_{1,2} \\ r_{2,1} & r_{2,2} \end{bmatrix} \tag{S24}$$

and a regularisation parameter. In this case, it is reasonable to extend the Lasso regularisation (S16) by an additional lower bound

$$0 \leq C^- \leq \alpha \leq C^+ \leq 1,$$

where C^- and C^+ are some a priori minimal and maximal probability bounds for observing the first category of the labels.

Since the simplified problem includes only two categories, we can introduce auxiliary notation for characteristic functions $\chi_{t,g} \in \{0, 1\}$ such that

$$\begin{aligned} \chi \left(Y^{\text{obs}}_{t,g} = y_1 \right) &= \chi_{t,g}, \\ \chi \left(Y^{\text{obs}}_{t,g} = y_2 \right) &= 1 - \chi_{t,g}. \end{aligned}$$

Using this, the objective function (S17) with (S21) can be written in the form (omitting index $g = 1$)

$$L(\alpha, C, r) = \frac{1}{T} \sum_{t=1}^{T} \left[\chi_t \log(\phi_1 r_{1,1} + \phi_2 r_{2,1}) + (1 - \chi_t) \log(\phi_1 r_{1,2} + \phi_2 r_{2,2}) \right]. \tag{S25}$$

Substituting the constant model (S23) and the equality constraints (S18), i.e.,

$$r_{2,1} = 1 - r_{1,1}, \;\; r_{1,2} = 1 - r_{2,2},$$

and using the following notation for the empirical relative frequency from the data statistics

$$N_T := \frac{1}{T} \sum_{t=1}^{T} \chi_t,$$

one can simplify (S25) even further, obtaining

$$\begin{aligned} L(\alpha, C, r) &= \frac{1}{T}\sum_{t=1}^{T}\left[\chi_t \log(\alpha r_{1,1} + (1-\alpha)(1-r_{1,1})) + (1-\chi_t)\log(\alpha(1-r_{2,2}) + (1-\alpha)r_{2,2})\right] \\ &= \frac{1}{T}\sum_{t=1}^{T}\left[\chi_t \log(1 - r_{1,1} - \alpha + 2\alpha r_{1,1}) + (1-\chi_t)\log(r_{2,2} + \alpha - 2\alpha r_{2,2})\right] \\ &= N_T \log(1 - r_{1,1} - \alpha + 2\alpha r_{1,1}) + (1 - N_T)\log(r_{2,2} + \alpha - 2\alpha r_{2,2}). \end{aligned} \tag{S26}$$

Finally, the simplified optimisation problem with unknown variables $\alpha, r_{1,1}, r_{2,2}$ is given by

$$L(\alpha, C, r) = N_T \log(1 - r_{1,1} - \alpha + 2\alpha r_{1,1}) + (1 - N_T)\log(r_{2,2} + \alpha - 2\alpha r_{2,2}), \tag{S27}$$

$$0 \le r^- \le r_{1,1} \le r^+ < 1, \tag{S28}$$

$$0 \le r^- \le r_{2,2} \le r^+ < 1, \tag{S29}$$

$$0 \le C^- \le \alpha \le C^+ \le 1. \tag{S30}$$

The problem (S27) can be considered as an extension of the well-known Bernoulli trials problem - by including the possibility of a random mislabeling in the data. It is easy to check that if we set the probability of correct labeling to 1 and the probability of mislabeling to 0 in (S24), i.e.,

$$\begin{aligned} \left[Y_{t,g} = y_1 | Y_{t,g}^{\text{obs}} = y_1\right] &= \left[Y_{t,g} = y_1 | Y_{t,g}^{\text{obs}} = y_1\right] = 1, \\ \left[Y_{t,g} = y_1 | Y_{t,g}^{\text{obs}} = y_2\right] &= \left[Y_{t,g} = y_2 | Y_{t,g}^{\text{obs}} = y_1\right] = 0, \end{aligned} \qquad r = \begin{bmatrix} 1 & 0 \\ 0 & 1 \end{bmatrix} = I,$$

and choosing $C^- = 0, C^+ = 1$, then the objective function (S27) is simplified to

$$L(\alpha, C, I) = N_T \log \alpha + (1 - N_T)\log(1 - \alpha)$$

and the solution of the respective (unconstrained) log-likelihood maximisation problem is given by

$$\phi_1 = N_T, \quad \phi_2 = 1 - N_T.$$

On top of this, an extended problem (S27,S28,S29,S30) includes an additional possibility of random mislabeling in the data. The following Lemma summarises the basic theoretical results.

Let $N_T \in (0, 1)$,

$$C^-, C^+ \in [0, 1], C^- \le C^+ \quad \text{and} \quad r^-, r^+ \in (0.5, 1), r^- \le r^+$$

be given. Then the optimisation problem (S27,S28,S29,S30) is well-defined and always has a solution.

a) Let $r_{1,1}, r_{2,2} \in [r^-, r^+]$ be given and fixed. Then

$$\alpha = \begin{cases} C^- & \text{if } \tau \le C^- \\ C^+ & \text{if } \tau \ge C^+ \\ \tau & \text{elsewhere} \end{cases} \qquad \tau := (1 - N_T)\left(1 + \frac{r_{1,1}}{1 - 2r_{1,1}}\right) - N_T \frac{r_{2,2}}{1 - 2r_{2,2}}$$

is a solution of the optimisation problem.

b) Additionally suppose that $r_{1,1} = r_{2,2} = \hat{r}$. If the set

$$\Omega_{N_T} := \{[\alpha, \hat{r}] \in [0,1]^2 : \hat{r} - 2\alpha\hat{r} + \alpha = 1 - N_T\} \cap \left([C^-, C^+] \times [r^+, r^-]\right) \tag{S31}$$

is non-empty, then it forms the system of all solutions of the optimisation problem.

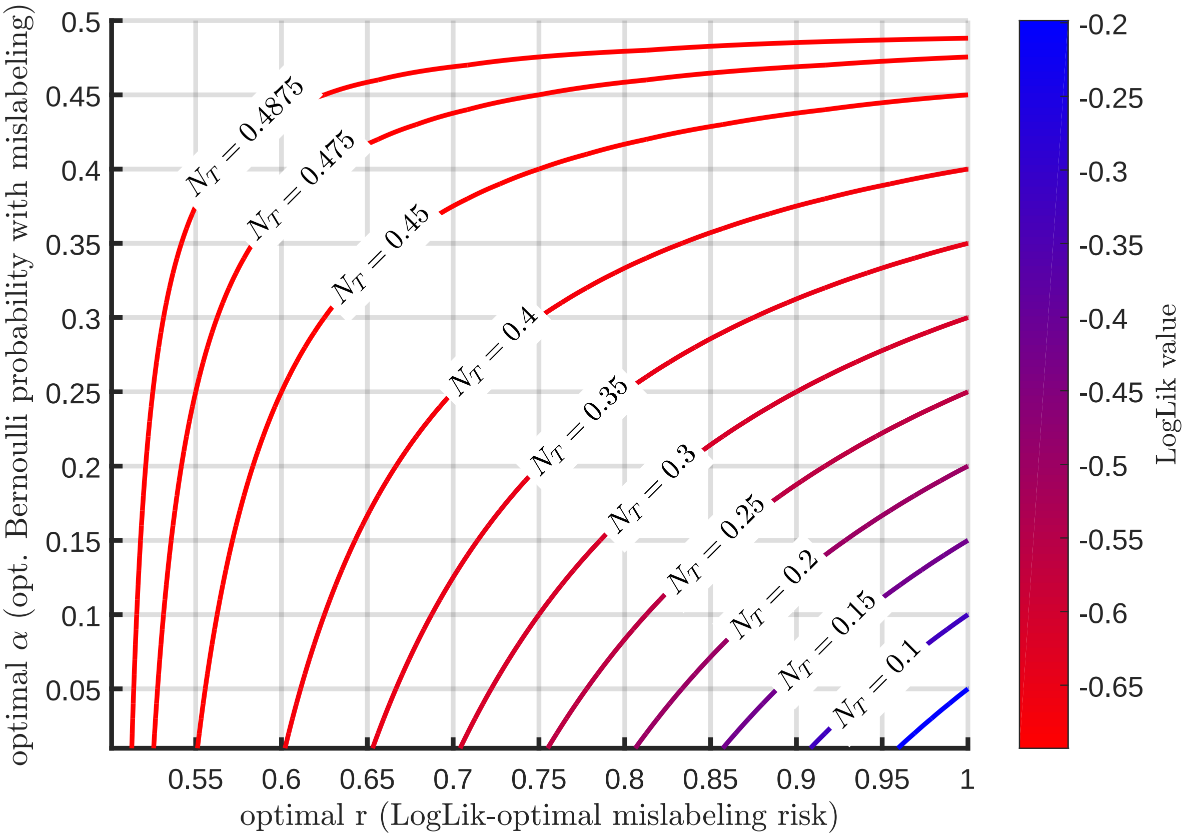

Figure S1. Graphical representation of the solution of to the Bernoulli trial problem with mislabeling using Lemma 2. The curves are the graphical representation of the solution set (S31) for various values of relative frequency N_T. The colour represents the final values of the objective Log-Likelihood function (S27).

Separation of variables:

From a numerical point of view, the problem (S22) for fixed $C = \hat{C}$ can be solved as a sequence of splitted two consecutive optimization problems, where we fix one variable and solve the problem with respect to the last remaining one. Such an approach generates the monotonically decreasing objective function, i.e. if for any given feasible initial approximation r^0 we construct the sequence of approximations solving for each $k = 0, 1, \dots$

$$\begin{array}{rcll} \alpha^{k+1} & = & \arg\min\limits_{\alpha} L\left(\alpha, \hat{C}, r^k\right) \text{ s.t. (S18),(S19)} & \text{(i.e. (S22) with fixed } C = \hat{C} \text{ and } r = r^k) \\ r^{k+1} & = & \arg\min\limits_{r} L\left(\alpha^{k+1}, \hat{C}, r\right) \text{ s.t. (S20)} & \text{(i.e. (S22) with fixed } C = \hat{C} \text{ and } \alpha = \alpha^{k+1}) \end{array} \tag{S32}$$

then using the optimality conditions of each inner optimization problem

$$\begin{array}{lrcl} \forall \alpha : & L\left(\alpha, \hat{C}, r^k\right) & \geq & L\left(\alpha^{k+1}, \hat{C}, r^k\right) \\ \forall r : & L\left(\alpha^{k+1}, \hat{C}, r\right) & \geq & L\left(\alpha^{k+1}, \hat{C}, r^{k+1}\right) \end{array} \tag{S33}$$

we have (choosing $\alpha = \alpha^k$ and $r = r^k$ in (S33))

$$L\left(\alpha^{k+1}, \hat{C}, r^{k+1}\right) \leq L\left(\alpha^{k+1}, \hat{C}, r^k\right) \leq L\left(\alpha^k, \hat{C}, r^k\right). \tag{S34}$$

The advantage of used splitting scheme is straightforward; the new optimisation problems have smaller dimension as well as they are easier for analysis. Additionally, the original problem (in both variables α and r) can be non-concave even if inner optimisation problems in one particular variable are concave. From a numerical point of view, convex (or concave) optimisation problem are generally easier to solve [1]. In the following text we focus on analysis of inner problems in (S32) separately.

Problem in variable r:

Let $\hat{\alpha}, \hat{C}$ be fixed feasible variables in problem (S17). Then the problem is given by

$$\mu(r) := L(\hat{\alpha}, \hat{C}, r), \quad \mu(r) \ \rightarrow \ \max \tag{S35}$$

with respect to constraints (S18), (S19).

Function $\mu(r)$ is concave on feasible set.

PROOF. Let Ω_r denote the convex feasible set determined by constraints (S18, S19). Based on the definition of concave function, it is necessary to prove that

$$\forall r_1, r_2 \in \Omega_r, \forall \sigma \in [0,1] : \quad (1-\sigma)\mu(r_1) + \sigma\mu(r_2) \leq \mu((1-\sigma)r_1 + \sigma r_2). \tag{S36}$$

The left side of this inequality can be rewritten using the definition of objective function (S17)

$$\begin{array}{rcl} (1-\sigma)\mu(r_1) + \sigma\mu(r_2) & = & \frac{1}{N_g T_g} \sum\limits_{g=1}^{N_g} \sum\limits_{t=1}^{T_g} \left[(1-\sigma) \log\left(\sum\limits_{i=1}^{m} \phi_i\left(X_{t,g}, \alpha\right) r_{1,i,\hat{\jmath}_{t,g},g} \right) \right. \\ & & \left. + \ \sigma \log\left(\sum\limits_{i=1}^{m} \phi_i\left(X_{t,g}, \alpha\right) r_{2,i,\hat{\jmath}_{t,g},g} \right) \right]. \end{array} \tag{S37}$$

Please notice that the logarithm is a concave function, i.e.,

$$\forall x_1, x_2 \in (0,\infty), \forall \sigma \in [0,1]: \quad (1-\sigma)\log(x_1) + \sigma \log(x_2) \leq \log((1-\sigma)x_1 + \sigma x_2),$$

therefore each of the terms in summation (S37) can be estimated from above

$$\begin{aligned}(1-\sigma)\mu(r_1) + \sigma\mu(r_2) \quad &\leq \quad \tfrac{1}{N_g T_g} \sum_{g=1}^{N_g} \sum_{t=1}^{T_g} \log\left[\sum_{i=1}^{m} \phi_i\left(X_{t,g},\alpha\right)\left((1-\sigma)r_{1,i,\hat{j}_{t,g},g} + \sigma r_{2,i,\hat{j}_{t,g},g}\right)\right] \\ &= \mu((1-\sigma)r_1 + \sigma r_2).\end{aligned}$$

Inequality (S36) is proven, therefore μ is concave.

The problem is concave, however, the solution can be non-unique. To demonstrate this, let us focus on one particular term in summation (S17) with fixed indexes $\hat{t}, \hat{g}$

$$\log\left(\sum_{i,j=1}^{m} \phi_i\left(X_{\hat{t},\hat{g}}, \hat{\alpha}\right) r_{i,\hat{j}_{\hat{t},\hat{g}},\hat{g}}.\right) \tag{S38}$$

We can see, that all variables $r_{i,j,\hat{g}}, j \neq \hat{j}$ are eliminated from the given term. There is a possibility that those variables are eliminated also from all other terms, i.e. from whole objective function (S35). In this case, such variables are incorporated in the optimisation problem only in the form of separable constraints (S18), (S19). Their values can be arbitrary chosen such that the resulting point will be still from the feasible set, but the value of objective function remains unchanged. In practice, this means that if we consider in our model a category which has not been observed in the data, then corresponding coefficients are not well defined and the problem has an infinite number of solutions.

Another possibility of term elimination occurs if

$$\forall i: \ \phi_i(X_{\hat{t},\hat{g}}, \hat{\alpha}) = \frac{1}{m} \tag{S39}$$

in particular term (S38) in summation (S17) of indexes $\hat{t}, \hat{g}$. Indeed, if we substitute these coefficients into given term, we obtain

$$\begin{aligned}\log\left(\sum_{i,j=1}^{m} \phi_i\left(X_{\hat{t},\hat{g}}, \hat{\alpha}\right) r_{i,j,\hat{g}} \chi\left(Y^{\text{obs}}_{\hat{t},\hat{g}} = y_j\right)\right) \quad &= \quad \log\left(\tfrac{1}{m} \sum_{i,j=1}^{m} r_{i,j,\hat{g}} \chi\left(Y^{\text{obs}}_{\hat{t},\hat{g}} = y_j\right)\right) \\ &= \quad \log\left(\tfrac{1}{m} \sum_{j=1}^{m} \left[\chi\left(Y^{\text{obs}}_{\hat{t},\hat{g}} = y_j\right) \underbrace{\sum_{i=1}^{m} r_{i,j,\hat{g}}}_{=1}\right]\right) \\ &= \quad \log\left(\tfrac{1}{m} \underbrace{\sum_{i=1}^{m} \chi\left(Y^{\text{obs}}_{\hat{t},\hat{g}} = y_j\right)}_{=1}\right) = \log\tfrac{1}{m}\end{aligned}$$

One can see that the reasons for the first and the second type of elimination are not dependent, however, together they are composing a priori unpredictable conditions for an infinite number of solutions.

Problem in variable α:

We fix $\hat{C}$ and $\hat{r}$ and we are interested in properties of the problem

$$\Psi(\alpha) := L(\alpha, \hat{C}, \hat{r}) = \frac{1}{N_g T_g} \sum_{g=1}^{N_g} \sum_{t=1}^{T_g} \log \left(\sum_{i=1}^{m} \phi_i \left(X_{t,g}, \alpha \right) \hat{r}_{i,\hat{j}_{t,g},g} \right), \quad \Psi(\alpha) \ \rightarrow \ \max \tag{S40}$$

with respect to constraint (S20). It is easy to prove (similarly to the proof of Lemma 2) that if ϕ_i are concave functions, then $\Psi(\alpha)$ is also concave function. However, concavity is not a typical property of standard probabilistic model functions. Those functions are typically log-concave, which is not a sufficient condition for concavity of $\Psi(\alpha)$ (convex linear combination of log-concave functions is generally not concave [1]).

Instead of solving (S40) directly, one can solve a so-called *relaxed* approximated problem, where the lower bound of the objective function is minimised. This approach is typical for several methods behind Expectation-Maximisation algorithms [8] (e.g., for Bayesian mixture models, Hidden Markov Models [8], and in Probabilistic Latent Semantic Analysis [6]), where instead of working with the original log-likelihood problem one creates the numerical maximisation scheme for its Jensen-approximated lower bound. In our case, the lower bound can also be obtained using Jensen inequality [9]; the logarithm is a concave function therefore one can estimate

$$\Psi(\alpha) \geq \frac{1}{N_g T_g} \sum_{g=1}^{N_g} \sum_{t=1}^{T_g} \sum_{i=1}^{m} \hat{r}_{i,\hat{j}_{t,g},g} \log \left(\phi_i \left(X_{t,g}, \alpha \right) \right) = \tilde{\Psi}(\alpha) \ \rightarrow \ \max . \tag{S41}$$

Since $\tilde{\Psi}(\alpha)$ is composed as a linear combination of concave functions (we suppose that $\phi_i(\alpha)$ are log-concave functions, therefore $\log \phi_i(\alpha)$ are concave functions) with non-negative coeficients, the function is concave itself. Additionally, if $\phi_i(\alpha)$ are strictly log-concave (which is for example the case for a logit function), then $\tilde{\Psi}(\alpha)$ is strictly concave and the solution of relaxed optimisation problem (S41) is unique.

In this paper, we discuss the solvability of the original problem (S40). Suppose the simplest case when $m = 2$ (the problem solved in this paper) and let $\phi_i(X_{t,g}, \alpha)$ be quasiconcave functions in variable α, i.e.,

$$\forall \alpha_1, \alpha_2, \forall \sigma \in [0,1] : \psi(\sigma\alpha_1 + (1-\sigma)\alpha_2) \geq \min\{\psi(\alpha_1), \psi(\alpha_2)\}. \tag{S42}$$

Let us remark that log-concave functions are quasiconcave [1]. We show that each term in sum (S40) is a monotonic function, however, in the case of strictly monotonic logarithm, it is sufficient to prove the monotonicity of argument of logarithm. Following Lemma presents the result.

Let ϕ_2 be a quasiconcave function such that $\forall \alpha : \phi_2(\alpha) \in [0,1]$. Let ϕ_1 be function such that $\forall \alpha : \phi_1(\alpha) + \phi_2(\alpha) = 1, r_1, r_2 \in [0,1], r_1 + r_2 = 1$. Then function

$$\psi(\alpha) = r_1 \phi_1(\alpha) + r_2 \phi_2(\alpha) \tag{S43}$$

is monotonic (i.e., it is both quasiconcave and quasiconvex).

PROOF. At first, notice that using assumptions we can write the function in form

$$\psi(\alpha) = r_1(1 - \phi_2(\alpha)) + r_2\phi_2(\alpha) = r_1 + (r_2 - r_1)\phi_2(\alpha).$$

Let us consider general α_1, α_2 and define

$$\alpha_\sigma = \sigma\alpha_1 + (1 - \sigma)\alpha_2$$

for arbitrary $\sigma \in [0, 1]$. Without loss of generality we suppose that $r_2 \geq r_1$. Then we can estimate

$$\begin{aligned}\psi(\alpha_\sigma) \quad &\geq \quad r_1 + \underbrace{(r_2 - r_1)}_{\geq 0} \min\{\phi_2(\alpha_1), \phi_2(\alpha_2)\} \\ &= \min\{r_1 + (r_2 - r_1)\phi_2(\alpha_1), r_1 + (r_2 - r_1)\phi_2(\alpha_2)\} = \min\{\psi(\alpha_1), \psi(\alpha_2)\},\end{aligned}$$

which proves the quasiconcavity. Quasiconvexity can be proven in a siminar way. We say, that a function ψ is quasiconvex, if $-\psi$ is quasiconcave [1]. We suppose that $r_1 \geq r_2$. Then we can estimate

$$\begin{aligned}-\psi(\alpha_\sigma) \quad &\geq \quad -r_1 + \underbrace{(r_1 - r_2)}_{\geq 0} \min\{\phi_2(\alpha_1), \phi_2(\alpha_2)\} \\ &= \min\{-r_1 + (r_1 - r_2)\phi_2(\alpha_1), -r_1 + (r_1 - r_2)\phi_2(\alpha_2)\} = \min\{-\psi(\alpha_1), -\psi(\alpha_2)\}.\end{aligned}$$

We can conclude that (S40) is a sum of monotonic function. However, the sum of monotonic functions is generally not a monotonic function itself; the sum of quasiconvex functions is not generally quasiconvex and the sum of quasiconcave functions is not generally quasiconcave.

One possibility how to enforce the (quasi)concavity of the objective function is to introduce to introduce the so-called *Tikhonov regularisation* and solve regularised problem

$$\Psi_\epsilon(\alpha) = \Psi(\alpha) - \epsilon^2\rho(\alpha) \quad \to \quad \max, \tag{S44}$$

where ρ is a non-negative strictly convex function (for instance in lasso-regularisation $\rho(\alpha) = \|\alpha\|_1$) and $\epsilon^2 > 0$ is *regularisation* parameter. The following Lemma presents the solvability of new (unconstrained) regularised optimisation problem.

Let $f : \mathbb{R}^n \to \mathbb{R}$ be continuous function bounded from above, i.e., there exists $b \in \mathbb{R}$ such that $\forall x \in \mathbb{R}^n : f(x) \leq b$. Let $\rho : \mathbb{R}^n \to \mathbb{R}$ be coercive functional, i.e.,

$$\lim_{\|x\|_1 \to \infty} \rho(x) = \infty \tag{S45}$$

and $f_\epsilon(x) = f(x) - \epsilon^2\rho(x)$. Then optimisation problem $f_\epsilon(x) \to \max$ has a solution for any $\epsilon^2 > 0$.

PROOF. Investigating the asymptotical behaviour of f_ϵ using boundedness and (S45) we get

$$\lim_{\|x\|_1 \to \infty} f_\epsilon(x) \leq b - \epsilon^2 \lim_{\|x\|_1 \to \infty} \rho(x) = -\infty \;\; \to \quad \lim_{\|x\|_1 \to \infty} f_\epsilon(x) = -\infty$$

and we can conclude that f_ϵ always attains maximum on $\mathbb{R}^n$.

Let $\epsilon^2 > \log m$. Then the bjective function (S44) is strictly quasiconcave and consequently given maximisation problem has an unique solution.

Numerics:

In the previous section, we have demonstrated that the problem in variable α is non-concave, therefore even if the sequence generated by algorithm (S32) is monotonically decreasing (S34), the final numerical solution could be only the local maximum depending on initial approximation of r^0. The classical approach is to run the algorithm with many random initial approximations and the final global solution can be chosen with respect to the obtained objective function values in the computed local solutions.

However, if the dimension of variable r is not too large, the feasible domain defined by (S18) and (S19) can be discretized using a regular grid. The nodes of this grid represent different fixed values of variable r and for each of this fixed values it remains to solve the appropriate problem in remaining variable α.

In combination with discretisation of parameter C we obtain following algorithm:

(i) first, the rectangular domain spanning a domain of mislabeling matrix elements in (S19) and admissible values of C in (S20) is sampled (e.g., by means of a uniform equidistant grid) and

(ii) for every particular grid point (r_s, C_s) one deploys some standard gradient-based method (e.g., the interior-points method or the sequential quadratic programming [9]) to perform a constrained optimisation of (S17) subject only to a constraint (S20) for fixed values of r_s and C_s.

For example, when $m = 2$ and $N_g = 1$ (the case emerging in both of the application examples from the main manuscript) there will be only two independent parameters in r. Together with the scalar dimension for the regularisation constant C this will result in a 3D grid (r_s, C_s).

For every particular grid point value (r_s, C_s) the concave maximisation procedure can be performed independent from the other grid points - allowing for a higly-scalable implementation when the problems (S17,S20) for different s are solved in parallel and without a need for communication between the different problems. Parallel MATLAB implementation of this algorithmic procedure is available as a part of the toolbox of methods and is provided for open access over GitHub at `https://github.com/SusanneGerber/Mislabeling_Coinference/tree/master/Release/Mislabeling_Coinference`[1].

[1] Provided open-source package requires availability of the software licences for “Parallel Computing” and “Optimization” toolboxes that are part of the standard MATLAB “Student’s Package”.

3 PERFORMANCE ON SYNTHETICALLY MISLABELED MAMMOGRAPHY DATA

Table S1. shows the performance of different model types on the mammography dataset with various mislabeling rates. All models, despite the original one, were trained using the respective mislabeled dataset. The average prediction accuracy was calculated based on the original mammography dataset. Co-Inference outperforms a linear SVC and performs nearly on par with an state of the art SVC using an RBF kernel

Model	data mislabeled	Accuracy	AUC
SVM – linear	0.00%	0.772	0.8575
SVM – linear	0.50%	0.774	0.859
SVM – linear	1.00%	0.775	0.859
SVM – linear	2.00%	0.774	0.860
SVM – linear	4.00%	0.764	0.853
SVM – linear	5.00%	0.759	0.851
SVM – RBF	0.00%	0.815	0.889
SVM – RBF	0.50%	0.819	0.892
SVM – RBF	1.00%	0.821	0.892
SVM – RBF	2.00%	0.823	0.892
SVM – RBF	4.00%	0.829	0.897
SVM – RBF	5.00%	0.832	0.898
Co-inference	0.00%	0.818	0.877
Co-inference	0.50%	0.812	0.873
Co-inference	1.00%	0.813	0.867
Co-inference	2.00%	0.808	0.860
Co-inference	4.00%	0.81	0.837
Co-inference	5.00%	0.813	0.842

4 LIST OF SNPS RELEVANT FOR CAUCASIAN WELLDERLY

-1 refers to the minor, 0 to mixed and 1 to a major allele.

- mean impact of (rs2112464 is -1) on Wellderly-probability is -0.30611;
- mean impact of (rs62087156 is -1) on Wellderly-probability is 0.28732;
- mean impact of (rs283753 is -1) on Wellderly-probability is 0.27068;
- mean impact of (rs60311669 is 1) on Wellderly-probability is 0.24601;
- mean impact of (rs75422555 is 1) on Wellderly-probability is -0.18855;
- mean impact of (rs1339899 is 1) on Wellderly-probability is -0.17661;
- mean impact of (rs306083 is 0) on Wellderly-probability is 0.16526;
- mean impact of (rs11741244 is 1) on Wellderly-probability is -0.13878;
- mean impact of (rs113874343 is 1) on Wellderly-probability is -0.13872;
- mean impact of (rs9930761 is 1) on Wellderly-probability is 0.12943;
- mean impact of (rs2740763 is -1) on Wellderly-probability is 0.12884;
- mean impact of (rs7798774 is 1) on Wellderly-probability is 0.12714;
- mean impact of (rs2237363 is 0) on Wellderly-probability is -0.12129;
- mean impact of (rs8008750 is 1) on Wellderly-probability is -0.11345;
- mean impact of (rs2081879 is 1) on Wellderly-probability is -0.1089;
- mean impact of (rs4791034 is 1) on Wellderly-probability is -0.10437;
- mean impact of (rs2946390 is 1) on Wellderly-probability is -0.098397;

- mean impact of (rs11834113 is 1) on Wellderly-probability is -0.097609;
- mean impact of (rs112299761 is 1) on Wellderly-probability is -0.096565;
- mean impact of (rs4298422 is 0) on Wellderly-probability is -0.091988;
- mean impact of (rs429358 is 1) on Wellderly-probability is 0.085594;
- mean impact of (rs2737628 is 0) on Wellderly-probability is -0.084257;
- mean impact of (rs84460 is -1) on Wellderly-probability is 0.082506;
- mean impact of (rs55931227 is 0) on Wellderly-probability is 0.08196;
- mean impact of (rs4915195 is 1) on Wellderly-probability is -0.076019;
- mean impact of (rs3020304 is -1) on Wellderly-probability is 0.070281;
- mean impact of (rs2504065 is -1) on Wellderly-probability is 0.0689;
- mean impact of (rs1121276 is 0) on Wellderly-probability is -0.067733;
- mean impact of (rs16035 is 0) on Wellderly-probability is -0.066701;
- mean impact of (rs11589267 is 0) on Wellderly-probability is 0.055175;
- mean impact of (rs28573147 is 0) on Wellderly-probability is -0.053381;
- mean impact of (rs4441745 is 0) on Wellderly-probability is 0.05153;
- mean impact of (rs2941741 is 0) on Wellderly-probability is -0.04984;
- mean impact of (rs77651534 is 1) on Wellderly-probability is -0.043252;
- mean impact of (rs4722782 is 1) on Wellderly-probability is -0.04026;
- mean impact of (rs11630259 is 1) on Wellderly-probability is 0.031974;
- mean impact of (rs762624 is 0) on Wellderly-probability is 0.031756;
- mean impact of (rs2536058 is 0) on Wellderly-probability is 0.031524;
- mean impact of (rs5746094 is 1) on Wellderly-probability is 0.027616;
- mean impact of (rs1121276 is -1) on Wellderly-probability is 0.027579;
- mean impact of (rs2854117 is 0) on Wellderly-probability is -0.023039;
- mean impact of (rs3774968 is 0) on Wellderly-probability is -0.022787;
- mean impact of (rs9900495 is 1) on Wellderly-probability is -0.0075964;
- mean impact of (rs4342445 is 1) on Wellderly-probability is 0.0039541;
- mean impact of (rs77651534 is 0) on Wellderly-probability is 0.0029776;

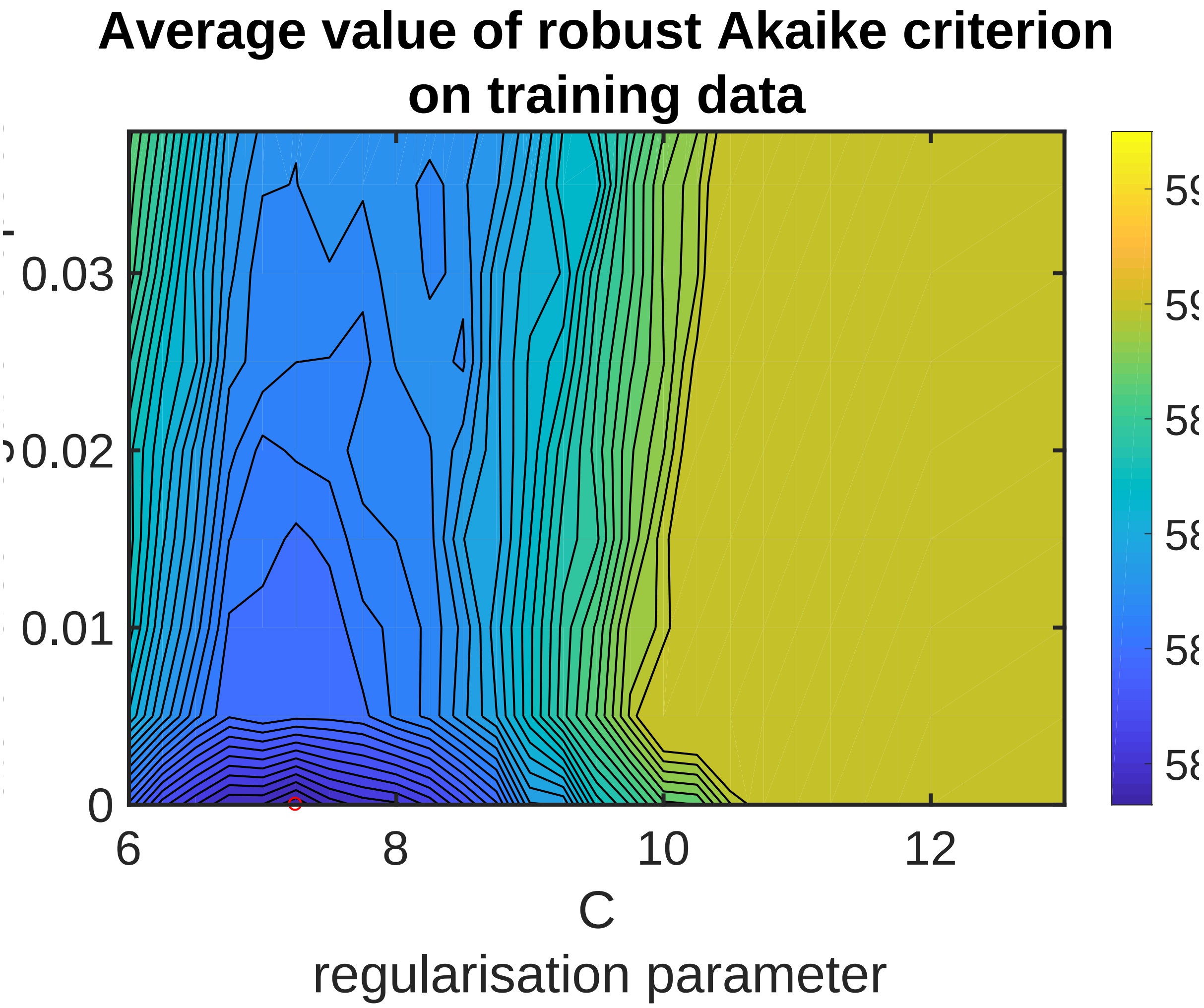

Figure S2. Example 1 from the main manuscript (breast cancer data analysis): model 5 selection by the robust Akaike Information criterion (AICc) [2], bootstrap-averaged values of the AICc on the training sets (with 500 bootstrap realisations).

REFERENCES

[1]Stephen Boyd and Lieven Vandenberghe. *Convex Optimization*. Cambridge University Press, New York, 1st edition, 2004.

[2]K.P. Burnham and D.R. Anderson. *Model selection and multimodel inference: a practical information-theoretic approach*. Springer-Verlag, 2002.

[3]Jerome Friedman, Trevor Hastie, and Robert Tibshirani. Regularization paths for generalized linear models via coordinate descent. *Journal of Statistical Software*, 33(1):1–22, 2010.

[4]J. Hadamard. Sur les problèmes aux dérivées partielles et leur signification physique. *Princeton University Bulletin*, 13:49–52, 1902.

[5]Trevor Hastie, Robert Tibshirani, and Jerome Friedman. *The elements of statistical learning: data mining, inference and prediction*. Springer, 2 edition, 2009.

[6]Thomas Hofmann. Probabilistic latent semantic indexing. *SIGIR Forum*, 51(2):211–218, August 2017.

[7]D. McFadden. Conditional logit analysis of qualitative choice behaviour. In P. Zarembka, editor, *Frontiers in Econometrics*, pages 105–142. Academic Press, NewYork, 1974.

[8]G.J. McLachlan and T. Krishnan. *The EM algorithm and extensions*. Wiley series in probability and statistics. Wiley-Interscience, 2008.

[9]J. Nocedal and S. J. Wright. *Numerical Optimization*. Springer, New York, 2nd edition, 2006.

[10]Noah Simon, Jerome Friedman, Trevor Hastie, and Rob Tibshirani. Regularization paths for cox's proportional hazards model via coordinate descent. *Journal of Statistical Software*, 39(5):1–13, 2011.

[11]Robert Tibshirani. Regression shrinkage and selection via the lasso. *Journal of the Royal Statistical Society. Series B (Methodological)*, 58:267–228, 1996.
